# Society Meetings

**Published:** 1904-07

**Authors:** 


### SOCIETY MEETINGS.

The American Association of Obstetricians and Gynecologists will hold its seventeenth annual meeting at the Hotel Monticello, Saint Louis, Tuesday, Wednesday, Thursday and Friday, September 13, 14, 15 and 16, 1904, under the following administration: president, Walter Blackburn Dorsett, Saint Louis; vice-presidents, Aaron B. Miller, Syracuse, and William D. Haggard, Nashville; secretary, William Warren Potter, Buffalo; treasurer, Xavier O. Werder, Pittsburg; executive council, Edwin Ricketts, Walter B. Chase, A. Vander Veer, Lewis S. McMurtry, L. H. Dunning and Rufus B. Hall.

The Hotel Monticello has been selected for the headquarters of the association, the management of which should be addressed concerning rooms and rates.

List of papers offered to June 28, 1904:

1. President's address, Walter B. Dorsett, Saint Louis.
2. Retrodisplacements as a cause of sterility; report of pregnancies following the Alexander operation, Herman E. Hayd, Buffalo.
3. Title to be announced, J. Henry Carstens, Detroit.
4. Cyst adenoma of the pancreas, L. H. Dunning, Indianapolis.
5. Some clinical reasons for advising early operations for fibroid tumors of the uterus, Rufus B. Hall, Cincinnati.
6. Operative treatment for relief of painful menstruation in virgins, W. A. B. Sellman, Baltimore.
7. Title to be announced, E. Gustav Zinke, Cincinnati.
8. Title to be announced, J. J. Gurney Williams, Philadelphia.

9. Pseudomembranous tubercular peritonitis, H. W. Longyear, Detroit.

10. The treatment of acute perforated gastric ulcer, Henry Howitt, Guelph.

11. Shall we remove all fibroid tumors of the uterus upon diagnosis? Thomas B. Eastman, Indianapolis.

12. Scar of sigmoid mesentery the cause of spastic obstruction of the bowels; with report of three cases, Hugo O. Pantzer, Indianapolis.

13. Surgical treatment of cicatricial atresia of the vagina, Charles G. Cumston, Boston.

14. Title to be announced, John B. Deaver, Philadelphia.

15. The advantage of limiting artificial interference in obstetric practice, A. P. Clarke, Cambridge.

16. Uterine myomas, with specimens, Joseph H. Branham, Baltimore.

17. Use of antistreptococcic serum in septicemia and scarlatina, with case histories, A. G. Hamilton, Springfield, Neb.

18. An unusual case with many of the symptoms of appendicitis, Magnus A. Tate, Cincinnati.

19. Conservation of the natural resistance of the patient in surgical work, Robert T. Morris, New York.

20. Title to be announced, J. E. Sadlier, Poughkeepsie.

21. Skeleton of an ectopic fetus removed by vaginal cystotomy, William D. Haggard, Nashville.

22. Title to be announced, L. S. McMurtry, Louisville.

23. Title to be announced, Charles A. L. Reed, Cincinnati.

---

The American Institute of Homeopathy held its annual meeting at Niagara Falls, June 20-24, 1904. The registry bore the names of about 350 members. Dr. George Royal, of Des Moines, Ia., was elected president.

